# Potato Yield and Quality, Soil Chemical Properties and Microbial Community as Affected by Different Potato Rotations in Southern Shanxi Province, China

**DOI:** 10.3390/plants15010117

**Published:** 2026-01-01

**Authors:** Jing Liu, Jundong Shi, Yongshan Li

**Affiliations:** 1 Cotton Research Institute, Shanxi Agricultural University, Yuncheng 044000, China; taozi131803@126.com (J.L.);; 2 Key Laboratory of Potato Genetic Improvement and Germplasm Innovation in Shanxi Province, Shanxi Agricultural University, Taiyuan 030031, China

**Keywords:** potato, tuber yield, quality, soil fertility, bacterial and fungal community

## Abstract

Continuous potato monoculture leads to yield decline, soil degradation, and increased soil-borne disease incidence. This study evaluated the potential of crop rotation to mitigate these issues by examining its effects on potato performance, soil chemical properties, and soil microbial communities. A two-year field experiment (2023–2024) in southern Shanxi, China, compared three treatments: continuous potato planting (CK, control), potato rotated with summer maize (with maize straw incorporation, T1), and potato rotated with summer soybean (with soybean straw incorporation, T2). The results demonstrated that both T1 and T2 rotations significantly increased tuber yield by 18.39% and 20.69%, respectively, and improved the potato commodity rate by 19.67% and 10.39%, compared to CK. Rotations also enhanced tuber quality, significantly increasing the content of nitrogen (5.24–28.20%), phosphorus (14.68–34.86%), potassium (23.61–52.42%), crude protein (5.14–28.11%), vitamin C (6.67–20.0%), starch (20.0–28.82%), and dry matter (4.55–12.88%), while reducing sugar content. In addition, the soil quality markedly improved under rotation. The soil organic matter, available phosphorus, available potassium, and total nitrogen increased by 27.77–31.92%, 10.48–12.38%, 4.44–28.42%, and 3.98–16.13%, respectively. *Proteobacteria*, *Actinobacteriota*, *Acidobacteriota*, *Chloroflex*, *Firmicutes*, and *Myxococcota* were the predominant bacterial phyla and *Ascomycota*, *Mortierellomycota*, *Basidiomycota*, and *Chytridiomycota* were the predominant fungal phyla. Microbial community analysis revealed that T1 rotation affected the Chao1 index and the ACE, measures of the diversity of the soil fungal community, and the rotations altered community structure. The abundance of pathogenic fungi, including *Fusarium*, *Alternaria*, and *Lectera*, was significantly reduced. Redundancy analysis (RDA) revealed that pH and total nitrogen (TN) were the primary factors shaping soil bacterial and fungal community structure. In conclusion, rotating potato with summer maize or soybean, combined with straw incorporation, is an effective strategy for enhancing tuber yield and quality, improving soil fertility, suppressing soil-borne pathogens, and promoting sustainable potato production in southern Shanxi.

## 1. Introduction

Potato (*Solanum tuberosum* L.) is the fourth most important food crop and the leading non-grain food in terms of global production, ranking after wheat, maize, and rice in 2023 [1,2] and it is planted worldwide. China widely cultivates potato and accounts for 24.45% of global potato productivity, with 27.21% of the global potato planting area in 2023 [1]. Because of its high yield potential and nutritional value, the potato is a crucial crop for enhancing global food security and dietary nutrition [3]. However, increasing pressure on arable land and the crop’s economic value have led to the widespread adoption of intensive, continuous potato monocropping [4]. This practice often results in degradation of soil fertility, imbalance of nutrients and microbes, serious soil-borne diseases, and potato yield reduction [5,6,7]. To mitigate these challenges, crop rotation has been established as a safe and effective agricultural strategy [8,9,10,11]. Numerous studies demonstrate that crop rotation can increase potato tuber yields [8,9,11,12,13,14], decrease negative effects on quality [8,15], and improve soil fertility [6]. For instance, Wang et al. reported that potato rotations with pea or faba bean significantly improved soil fertility and microbial quantity, improved enzyme activity, and increased tuber yield by 21.19% and 28.38% compared to that of continuous potato cropping [11]. Similarly, Xie et al. revealed that various crop rotations increased potato yield by 15.0–38.2%, improved net economic benefits by 30.6–41.9%, and enhanced the soil health index by 13.1–63.4%, compared with continuous potato cropping [16]. However, the specific benefits are highly dependent on the crop rotation patterns employed, leading to varied effects on the potato yield and soil nutrient status [11,12,16,17].

The efficacy of crop rotation is largely mediated through its impact on soil microbial communities, which are fundamental to maintaining soil quality, ecosystem functions, and nutrient cycling. A prevailing observation is that long-term potato monoculture shifts the soil microbial composition from bacterial to fungal dominance [18,19,20]. Research findings on how continuous potato cropping affects soil microbes have been inconsistent. Some studies indicate that potato monocropping decreased bacterial community diversity and richness with no significant effect on fungal communities, alongside an increased abundance of pathogenic fungi like *Fusarium* that correlate with disease incidence and yield decline over time in Gansu Province, China [21]. Qin et al. revealed that continuous potato planting reduced the bacterial community diversity and the abundance of beneficial bacteria in the soil by employing a T-RFLP technique in Yunnan, China [22]. Different crop rotations have obvious effects on soil microbial communities [23]. Qin et al. and Tan et al. reported that potato rotation decreased the bacterial community diversity and increased the fungal community in clay loam in Hebei Province [24] and in black soil in Gansu Province, China [25]. Techniques such as BIOLOG profiling have shown that rotations with barley and sweet corn support higher microbial functional diversity, unlike soybean–potato rotations on Bangor silt loam in the USA [23]. Ma et al. reported that potato–summer maize rotations changed the soil microorganism community structures on purple loam in Sichuan Province, China [26]. Nanak et al. revealed that potato–mung bean rotations had higher microbial biomasses of N and C in Pakistan [27].

Soil microorganisms are crucial for plants’ health. Beneficial and harmful microorganisms coexist in the soil. Their ratios determine plant health status and yield. Potato yield losses were caused by many diseases, such as black scurf (caused by *Rhizoctonia solani*), common scab (caused by *Streptomyces scabies)*, and Verticillium wilt (caused by *Verticillium dahliae*) [28,29]. Crop rotation can modulate the fungi-to-bacteria ratio [30] and actively reshape the overall microbial community structure [24,31,32]. Rotation decreased the abundance of harmful microorganisms such as *Fusaria* and *Mortierella* [33], creating negative effects on plant root growth [34]. Liu et al. found that abundance of *Fusarium* increased linearly over time and was closely associated with yield decline [21]. Microbial C and N had a positive relationship with biological and tuber yield [27]. Hemkemeyer et al. found that potato yield and quality were related with cover crop and soil microbiome [8]. Therefore, potato rotation is necessary to improve the productivity and sustainability of potato production.

Potato achieves optimal productivity under cool and frost-free conditions, rather than hot conditions [35]. Consequently, adapting planting dates and selecting suitable cultivars are critical management strategies [36,37,38]. The study area, Yuncheng in southern Shanxi, experiences a warm climate, especially after June, and the high temperature is not conducive to the growth of potatoes. To overcome this challenge and to capitalize on early market opportunities, local practices involve early planting dates with plastic mulching using early-maturing potato varieties, leading to harvests in early June [39]. This strategy provides a significant economic advantage over that of the adjacent regions that rely on autumn/winter production dominated by medium- to late-maturing varieties. The region belongs to the double-cropping system. Summer maize and summer soybean are traditional staple crops. After early potato harvests in early June, summer maize and summer soybean are a good fit for the planting schedule. Potato rotations with summer maize and soybean can mitigate continuous planting issues and improve yield and economic income. However, the specific impacts of crop rotation on potato yield, tuber quality, soil properties, and the microbial community within this unique, high-value, early-season production system are not well understood. Therefore, the objectives of this study were as follows: (1) evaluate the effects of rotations on potato tuber yield and quality; (2) assess the changes in key soil chemical properties under rotation systems; (3) determine the response of the soil bacterial and fungal community to rotation.

## 2. Results

### 2.1. Potato Tuber Yield and Quality

The different potato rotations significantly influenced the number of tubers per plant, the tuber weight/plant, the commodity rate, the tuber size distribution, and the total tuber yield (Table 1). Compared to the continuous cropping control (CK), both rotation treatments (T2 and T1) significantly increased the total tuber yield, by 20.69% and 18.39%, respectively (*p* < 0.05). Although the yield in T2 was 1.94% higher than in T1, this difference between the two rotation treatments was not statistically significant (*p* > 0.05). At the plant level, rotation led to substantial improvements: the number of tubers increased by 28.57% to 42.86% and the tuber weight per plant significantly increased by 14.94% to 26.44%, compared to CK, respectively. Furthermore, rotation altered the size distribution of the tubers favorably. The proportion of large tubers increased by 16.72% (T1) and 22.54% (T2), while the proportion of medium-sized tubers increased by 20.86% (T1) and 4.90% (T2), whereas the small potato rate significantly decreased by 30.02% in T2 treatment and 56.80% in T1 treatment (*p* < 0.05), compared to CK.

Different potato rotation systems significantly influenced the potato quality parameters (Table 2). Compared to the continuous cropping control (CK), the T2 treatment led to significant increases in tuber nitrogen (N), phosphorus (P), and potassium (K) content, of 28.20%, 34.86%, and 52.42%, respectively (*p* < 0.05). The T1 treatment resulted in a non-significant increase in N (5.24%) and P (14.68%) content (*p* > 0.05), but a significant increase in K content (23.61%; *p* < 0.05). Rotation treatments also significantly affected other nutritional components. The crude protein content increased significantly by 28.11% in T2 (*p* < 0.05), while the 5.14% increase in T1 was not significant. Similarly, vitamin C content saw a significant 20.0% increase in T2 (*p* < 0.05), compared to a non-significant 6.67% increase in T1. In contrast, starch content increased significantly under both rotation systems, by 28.82% in T1 and 20.0% in T2 (*p* < 0.05). The reducing sugar content decreased significantly by 3.01% in T2, but the 1.20% decrease in T1 was not significant. Finally, the dry matter content increased significantly by 12.88% in T2 (*p* < 0.05), with T1 showing a non-significant increase of 4.55%.

### 2.2. Soil Chemical Properties

The effects of different potato rotations on the soil physicochemical properties are summarized in Table 3. Compared with CK, T2 treatment significantly decreased soil pH (*p* < 0.05), whereas the pH in the T1 treatment was not significantly different from CK. Both rotation treatments significantly enhanced soil fertility. Soil organic matter (OM) significantly increased by 19.12% in the T2 treatment and 10.96% in the T1 treatment, compared with CK, respectively. For soil available nutrients, T2 significantly increased available phosphorus (AP) by 12.38% and available potassium (AK) by 17.65% (*p* < 0.05), while T1 showed non-significant increases of 3.01% in available nitrogen (AN) and 5.83% in AK (*p* > 0.05). The increases in AN for T1 and T2 were not statistically significant compared to CK (*p* > 0.05). Similarly, soil total nitrogen (TN) significantly increased by 16.13% in T2 (*p* < 0.05), compared to a non-significant increase of 3.98% in T1.

### 2.3. Bacterial and Fungal Community Diversity with Different Rotation Treatments

**Figure 1:**
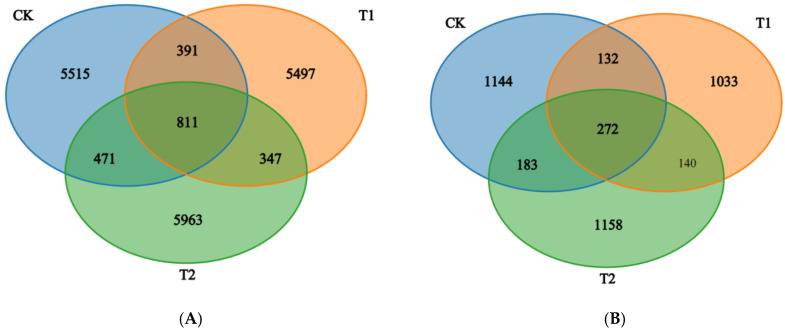
A Venn diagram of the number of shared and unique OTUs for the bacterial (**A**) and fungal (**B**) communities between different rotation treatments.

Analysis of the bacterial communities via MiSeq sequencing yielded a total of 18,995 operational taxonomic units (OTUs) across the nine soil samples, with 811 OTUs shared among all treatments (Figure 1A). T2 rotation resulted in a significantly higher number of bacterial OTUs compared with CK, whereas the OTU count in T1 was not significantly different from CK (*p* > 0.05; Figure 1). For fungal communities, a total of 4032 OTUs were obtained from the nine samples and 272 OTUs were shared between all soil samples (Figure 1B); The number of fungal OTUs across the treatments was ordered as follows: T2 > CK >T1.

Rotation of potato with summer maize and soybean had no significant impact on the diversity of the soil bacterial community (Table 4). For soil bacteria, the T2 treatment resulted in higher alpha diversity, as indicated by the increased ACE, Chao1, and Shannon indices compared to the control (CK). In contrast, the T1 treatment showed lower values for these indices relative to CK. For the soil fungal community, T2 treatments tended to have non-significant higher values of the ACE, Chao1, and Shannon indices, compared with CK. The Chao1 index and the ACE of the T1 treatment showed a significant difference to that of T2 and CK.

### 2.4. Bacterial and Fungal Composition and Structure with Different Rotation Treatments

**Figure 2:**
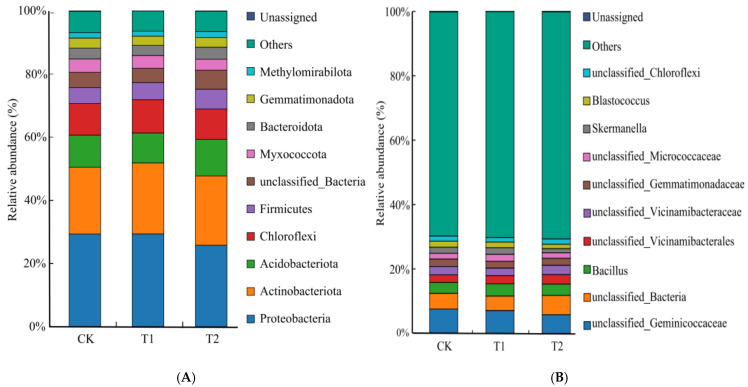
Soil bacterial relative abundance of different treatments at phylum (**A**) and genus (**B**) levels.

For the bacterial community, sequencing of bacterial OTUs identified a total of 41 phyla, 99 classes, 286 orders, 560 families, 1034 genus, and 1224 species across all samples. The different potato rotations altered the composition of the bacterial communities, particularly at the phylum and genus levels. At the phylum level, *Proteobacteria*, *Actinobacteriota*, *Acidobacteriota*, *Chloroflex*, *Firmicutes*, *unclassified_Bacteria*, *Myxococcota*, *Bacteroidota*, *Gemmatimonadota*, and *Methylomirabilota* were the 10 most abundant bacterial phyla in all samples (Figure 2A), of which *Proteobacteria*, *Actinobacteriota*, *Acidobacteriota*, *Chloroflexi*, and *Firmicutes* accounted for 71.10% to 80.26% of the total abundance. Rotation treatments also significantly altered the relative abundance of key phyla. Compared to both CK and T1, the T2 treatment significantly increased the relative abundance of *Acidobacteriota* by 14.45% and 22.53%, while significantly decreasing *Proteobacteria* by 12.1% and 12.19% (*p* < 0.05); no significant difference in these phyla was observed between T1 and CK. Finally, both T1 and T2 rotation significantly increased the relative abundance of *Actinobacteriota* by 6.34% and 3.64%, compared with CK, respectively. For *Firmicutes*, T1 and T2 rotation significantly increased its abundance by 8.68% and 25.53%, compared with CK, though the increase in T2 alone compared to CK was not statistically significant. The relative abundance of *Chloroflexi* increased by 5.29% in T1 rotation, but decreased by 4.10% in T2, compared to CK.

At soil bacterial genus levels, the 11 most abundant taxa were *unclassified_Geminicoccaceae*, *unclassified_Bacteria*, *Bacillus*, *unclassified_Vicinamibacteraceae*, *unclassified_Vicinamibacterales*, *unclassified_Gemmatimonadaceae*, *Skermanella*, *Blastococcus*, *unclassified_Micrococcaceae*, *unclassified_Chloroflexi*, *Rubrobacter*, *MND1*, and *RB41* in all samples (Figure 2B), collectively representing 33.04% to 34.29% of the total sequences. Rotation treatments also induced changes at the genus level. The relative abundance of *Bacillus* was significant, 12.38% higher in the T1 treatment and 4.47% higher in the T2 treatment (*p* > 0.05) than those in CK. For *Skermanella*, the abundance was significantly higher in the T1 treatment (8.45%), but significantly lower in the T2 treatment (32.45%) relative to CK. In contrast, the relative abundance of *Blastococcus*, *Rubrobacter,* and *MND1* was significant lower in the T1 and T2 treatments. The relative abundance of *RB41* was 10.55% lower in T1, but 13.30% higher in T2, than that of CK.

**Figure 3:**
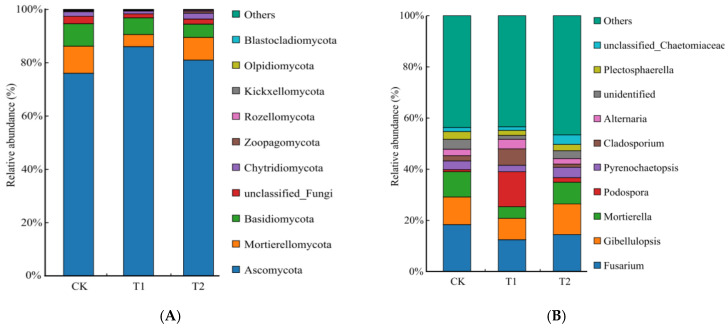
The soil fungal relative abundance of different treatments at phylum (**A**) and genus (**B**) levels.

For the fungal community, sequencing of fungal OTUs identified a total of 16 phyla, 50 classes, 113 orders, 251 families, 519 genus, and 793 species across all samples. The different potato rotations significantly altered the composition of fungal communities at both the phylum and genus levels (Figure 3). The ten most abundant fungal phyla were *Ascomycota*, *Mortierellomycota*, *Basidiomycota*, *unclassified_k__Fungi*, *Chytridiomycota*, *Zoopagomycota*, *Rozellomycota*, *Kickxellomycota*, *Olpidiomycota*, and *Blastocladiomycota* in all samples (Figure 3A). The three dominant phyla: *Ascomycota* (76.33–85.96%), *Mortierellomycota* (4.58–10.04%), and *Basidiomycota* (4.99–8.28%), collectively accounted for 94.52% to 96.85% of the total abundance.

The rotation treatments significantly altered the relative abundances of the dominant fungal phyla. Compared with CK, the relative abundance of *Ascomycota* significantly increased by 12.62% and 6.16% in the T1 and T2 treatments (*p* < 0.05). Conversely, *Mortierellomycota* and *Basidiomycota* significantly decreased in relative abundance in the rotation treatments, with reductions of 15.49–54.41% and 23.74–39.65%, respectively (*p*< 0.05). The response of *Chytridiomycota* was treatment-specific, showing a significant decrease of 37.23% in T1, but a significant increase of 27.87% in T2, relative to CK (*p* < 0.05).

The potato rotation treatments significantly altered the composition of fungal communities at a genus level (Figure 3B). *Fusarium*, *Gibellulopsis*, *Mortierella*, *Podospora*, *Pyrenochaetopsis*, *Cladosporium*, *Alternaria unidentified*, *Plectosphaerella*, and *Lectera* were the ten most abundant fungal genus in all samples, accounting for 49.70–55.13% of the total abundance.

The rotation treatments induced significant changes in the relative abundance of specific genera. Compared to the CK, the relative abundance of *Fusarium* was significantly lower in both the T1 (−31.39%) and T2 (−21.03%) treatments (*p* < 0.05). The genera *Gibellulopsis* and *Pyrenochaetopsis* exhibited contrasting responses. Their abundances were significantly lower in T1 (−23.39% and −25.05%, respectively) but significantly higher in T2 (+10.89% and +19.00%, respectively) compared to CK (*p* < 0.05). The relative abundance of *Mortierella* was significantly lower in the T1 treatment than in both the T2 and CK treatments (*p*< 0.05). Conversely, the relative abundance of *Podospora* was highest in the T1 treatment, showing significant differences compared to both the CK and T2 treatments (*p* < 0.05). The relative abundance of *Alternaria* significantly increased by 47.96% in the T1 treatment, but decreased by 14.17% in the T2 treatment, relative to CK, respectively (*p* < 0.05). Finally, the relative abundance of *Lectera* was significantly lower in both the T1 (−22.40%) and T2 (−28.19%) treatments compared with CK (*p* < 0.05).

**Figure 4:**
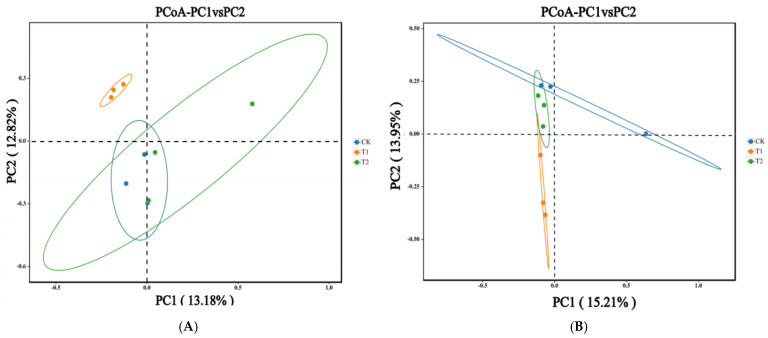
Principal coordinates analysis (PCoA) plots based on the Bray–Curtis distances of the bacterial (**A**) and fungal (**B**) communities at the OTU level (97% sequence similarity) in the different potato rotations. CK: Potato–potato rotation; T1: potato–summer maize rotation with maize straw incorporation; T2: potato–summer soybean rotation with soybean straw incorporation.

Different crop rotations significantly altered the soil microbial community structure. Principal coordinates analysis (PCoA) based on the binary Bray–Curtis distance, was used to visualize the differences in the rhizosphere bacterial and fungal community among potato rotation treatments (Figure 4). The first two principal coordinates explained 13.18% and 12.82% of the total variation in bacterial community composition, and 15.21% and 13.95% of the total variation in fungal community composition, respectively. The PCoA revealed a clear separation among treatments. For the bacterial community, the T1 treatment formed a distinct cluster separate from both the CK (control) and T2 treatments. In the fungal community, all three treatments (T1, T2, and CK) were distinctly separated from one another.

### 2.5. Soil Microbial Relationships with Soil Properties

**Figure 5:**
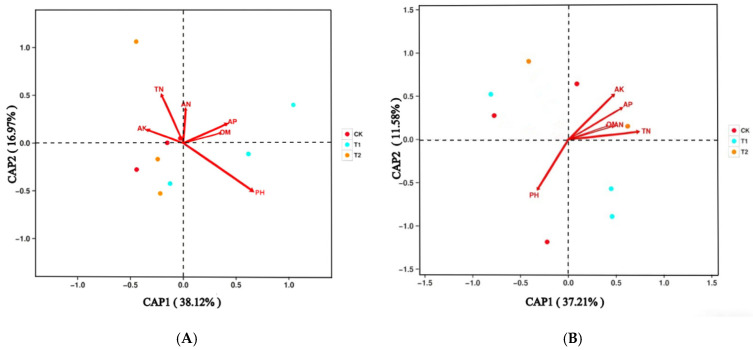
Redundancy analysis (RDA) of soil bacterial (**A**) and fungal (**B**) communities and soil characteristics. Different colors and symbols indicate different treatments. AN: Available nitrogen; AP: available phosphorus; AK: available potassium; TN: total nitrogen; OM: organic matter. CK: Potato–potato rotation; T1: potato–summer maize rotation with maize straw incorporation; T2: potato–summer soybean rotation with soybean straw incorporation.

Redundancy analysis (RDA) revealed that the first two axes explained 55.09% and 48.79% of the total variance in the soil bacterial and fungal community, respectively (Figure 5). For bacteria, pH and total nitrogen (TN) were the environmental factors most strongly correlated with the soil bacterial community structure, followed by available phosphorus (AP), available potassium (AK), available nitrogen (AN), and organic matter (OM). For fungi, the fungal community structure was significantly correlated with pH, TN, AK, and AP.

**Figure 6:**
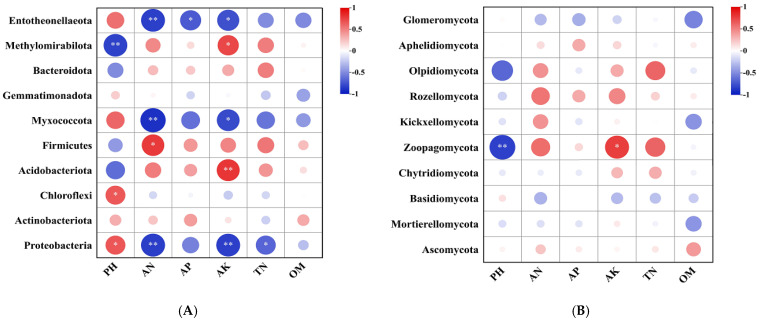
Relationships between soil chemical properties and bacterial (**A**) and fungal (**B**) phyla communities. * and ** indicate significance at the 0.05 and 0.01 levels, respectively. The size of the circle indicates the magnitude of the value.

Pearson’s correlation analysis revealed relationships between the dominant bacterial and fungal phyla and the soil chemical parameters (Figure 6). For bacteria, the relative abundance of *Proteobacteria* had a significant negative relationship with TN, AN, and AK, but was positively correlated with pH (*p* < 0.05). The relative abundances of *Chloroflexi* and *Acidobacteriota* were positively correlated with pH (*p* < 0.05) and AK (*p* < 0.01), respectively. The relative abundance of *Firmicutes* was positively correlated with AN (*p* < 0.05). The relative abundance of *Myxococcota* was negatively correlated with AN (*p* < 0.01) and AK (*p* < 0.05), respectively. The relative abundance of *Methylomirabilota* had a significant negative relationship with pH (*p* < 0.01) and a positive relationship with AK (*p* < 0.05). The relative abundance of *Entotheonellaeota* was correlated with AN, AP, and AK. Regarding the main phyla of fungi, the relative abundance of *Zoopagomycota* had a significant negative relationship with pH (*p* < 0.01) and a positive relationship with AK (*p* < 0.05). The other dominant microbes had no significant correlations with the soil properties.

## 3. Discussion

### 3.1. Effects of Rotation on Potato Tuber Yield and Quality

Many studies have demonstrated that various crop rotation patterns can significantly increase potato yield [27,33]. Consistent with this, our results indicate that potato tuber yield and quality were significantly affected by the rotation treatments (Table 1 and Table 2). Specifically, the T1 and T2 rotation treatments significantly increased potato tuber yield by 18.39% and 20.69%, respectively, compared to the control (*p* < 0.05). This increase can be attributed to a higher number of tubers per plant and greater tuber weight per plant. Our finding that rotation increases tubers per plant is consistent with previous reports with a 4-year rotation (potato–oat–faba bean–potato) and a seven-course crop rotation (potato–spring barley–alfalfa–alfalfa–spring oilseed rape–winter wheat–winter rye) [12,24], though it contrasts with the results of Mohr et al., who found that rotations of potato–canola–wheat or potato–canola–alfalfa–alfalfa affected tuber size distribution, but not the total tuber number [40].

The tuber yield in the T2 treatment was 1.94% higher than in T1, but this difference was not statistically significant. This aligns with studies by Nanak Khan et al. (potato–mung bean and potato–maize) [27] and others [30,41], which reported that legume-based rotations (like T2) generally produce higher tuber yields than non-legume rotations. For instance, Ma et al. documented a yield increase of 72.35% to 111.93% for a potato–summer corn rotation compared to continuous potato monocropping [26]. This contribution suggests that mineralization caused by plant residues increases the soil nutrient availability and leads to high yield [42]. The increase in potato tuber yield was attributed to the crop rotation, which improved the soil nutrient status, promoted plant growth, and increased the number of tubers and the yield.

Different crop rotation patterns significantly affected the potato quality. The commodity rate was significantly higher in the T1 and T2 rotations compared to the CK (control) treatment. This finding is consistent with the study by Carter et al., which reported clear differences in marketable yield between different crop rotations [43].

The nutrient concentrations generally followed the order T2 > T1 > CK. Compared to CK, the T2 rotation significantly increased N, P, and K concentrations by 28.20%, 34.86%, and 52.42%, respectively, (*p* < 0.05). The T1 rotation increased N and K concentrations by 14.68% (not significant) and 23.61% (*p* < 0.05), respectively, compared to CK. These results are supported by previous research. Ekeberg and Riley demonstrated that specific rotations increased tuber N, P, and K concentrations compared to conventional tillage [44]. Similarly, Jahanzad et al. reported that rotations incorporating cover crops (e.g., winter pea, rye, and forage radish) enhanced tuber yield and improved macro- and micronutrient content compared to systems without cover crops [45].

Beyond its high nutritional value, potato is an important crop for the food, starch, and alcohol industries. The key components such as the dry matter content, vitamin C, starch, and sugar-reducing content—which are critical for processing quality—are influenced by variety and agricultural practices [46].

A high dry matter content is desirable, particularly for fried products, as it minimizes fat absorption during processing [46]. In our study, tuber dry matter content was significantly higher in the T2 rotation than in the CK (control) treatment. Although the T1 rotation also had a higher mean value than CK, the difference was not statistically significant. This aligns with findings by Azimi et al., who reported that specific rotations, such as potato–soybean–barley, resulted in a higher starch and dry matter content compared to other sequences like potato–barley–red clover [47]. Furthermore, the contents of crude protein, vitamin C, and starch were significantly higher in the T2 rotation than in both the T1 and CK treatments.

Reducing sugar content is a critical quality parameter for processing, especially for frying. High concentrations lead to undesirable dark brown coloration in the final product, so a low content is essential [46]. In this study, both the T1 and T2 rotations resulted in a lower reducing sugar content compared to CK. These results are consistent with previous reports that crop rotation can increase the amount of desirable components like dry matter, starch, and vitamin C, while decreasing the reducing sugar content of potato tubers [46].

Overall, the chemical composition of potatoes is determined by many agroecological and production factors such as the soil, fertilization, and climatic conditions, as well as the genetic characteristics of the cultivar [48,49,50]. In this study, potato–summer soybean and potato–summer maize rotations improved soil nutrient status, promoted plant growth and nutrient uptake, and increased tuber yield and quality.

Although the agronomic benefits of the T2 treatment are clear, tuber yield and quality are ultimately the product of edaphic conditions and below-ground biota. The simultaneous improvement in soil fertility parameters (Section 3.2) and the shift toward a more suppressive microbial community (Section 3.3) under the T2 treatment therefore provides a mechanistic explanation for the gains reported here. Disentangling how the chemical and biological changes interact is the focus of the following sections.

### 3.2. Effects of Potato Rotation on Soil Chemical Properties

Studies have demonstrated that crop rotations can significantly enhance soil properties, improve soil health, and increase nutrient availability [6,11,16,24,26]. In this study, significant differences in soil properties were observed among the three treatments (Table 2). Soil pH in the T2 rotation was significantly lower than in the CK (control), while no significant difference was found between T1 and CK. The observed pH reduction can be attributed to the accumulation of the soil mineral N, a recognized factor that decreases soil pH [51].

Soil organic matter (OM) significantly increased by 19.12% in the T2 treatment and 10.96% in the T1 treatment, compared with CK (*p* < 0.05). Furthermore, both the T1 and T2 treatments resulted in higher levels of soil available nitrogen (AN), total nitrogen (TN), available phosphorus (AP), and available potassium (AK) than the CK treatment. These findings are consistent with previous research showing that the addition of organic nutrients can increase biological yields by 24.8% and improve soil organic carbon, available N, P, and K [52].

The improvement in soil fertility is primarily attributed to the addition of organic matter from the companion crops within the rotation system [53,54,55]. This is supported by studies such as Ananda et al., who reported that legume-based cropping systems accumulate higher soil carbon and nitrogen stocks than systems based solely on cereals [56]. As a legume, soybean can fix atmospheric N; its subsequent residue mineralization increases soil nutrient availability, which can lead to higher crop yields [42]. This mechanism explains why the T2 rotation, which included a legume, resulted in higher soil nutrient levels than the T1 rotation.

Taken together, the pronounced increases in available N, P, and SOM under T2 coincide with the 19% yield advantage and higher tuber-specific gravity documented in Section 3.1, indicating that enhanced nutrient supply is one (but not necessarily the only) driver of the rotation effect. The soil chemical shifts shown here may also have created a more favorable niche for beneficial microbes and a less hospitable environment for pathogens; evidence for this is presented in the following section on the soil microbiome.

### 3.3. Effects of Rotation on Soil Bacterial and Fungal Community

Crop rotation affected soil microbial diversity. The T2 rotation had higher Shannon, ACE, and Chao1 indices of bacterial community than CK and the T1 rotation had lower diversity than CK, but there was no significant difference. This was inconsistent with previous reports indicating that potato rotation with different oat and faba bean sequences decreases bacterial diversity [24]. This discrepancy may be attributed to soil type, as İnceoğlu et al. demonstrated that bacterial communities in the potato rhizosphere can vary substantially across different soil types, even when the same potato cultivar is used [57]. The Chao1 index and the ACE index of fungal community diversity in the T1 treatment was significantly lower than that of T2 and CK. These results did not align with Qin et al.’s previous findings that potato rotation increased fungal community diversity [24].

Soil microbiomes are influenced by crop species and genotype through the release of specific chemical compounds, which in turn alter soil organism composition and structure, ultimately affecting subsequent crop yield and quality [58]. Numerous studies have confirmed that different crop rotations significantly modify soil bacterial and fungal community composition and structure [6,11,16,24,26]. In this study, principal coordinates analysis (PCoA) showed that crop rotation significantly altered soil bacterial and fungal community structure (Figure 6).

In the soil bacterial community, in this study, *Proteobacteria*, *Actinobacteriota*, *Acidobacteriota*, *Chloroflex*, *Firmicutes*, *unclassified_Bacteria*, *Myxococcota*, *Bacteroidota*, *Gemmatimonadota, and Methylomirabilota* were the 10 most abundant bacterial phyla in all samples. The top five phyla (i.e., *Proteobacteria*, *Actinobacteriota*, *Acidobacteriota*, *Chloroflexi,* and *Firmicutes*) collectively accounted for 71.10% to 80.26% of the total abundance, a finding consistent with previous reports of potato–summer maize [26]. *Proteobacteria* was the dominant phylum in all treatments, aligning with its known predominance in potato-cultivated soils [21,24,32]. However, the T2 rotation had a significantly lower abundance of *Proteobacteria* compared to T1 and CK.

Shifts in the relative abundance of other key phyla were also observed. First, *Copiotrophic Phyla*: *Bacteroidetes*, *Gemmatimonas*, and *Proteobacteria* are considered copiotrophic bacteria, which thrive in nutrient-rich conditions by decomposing labile organic carbon [59,60]. The T2 rotation had a higher relative abundance of *Bacteroidetes*, while T1 had a lower abundance. Both the T1 and T2 rotations resulted in a lower relative abundance of *Gemmatimonadota*. Second, organic matter decomposers: the abundance of *Chloroflexi*, which is associated with the degradation of organic residues [61], was higher in T1 than in CK and T2. Third, soil health indicators: the abundance of *Firmicutes*, which can restrict soil pathogen growth and is associated with healthier soils [10], increased in both the T1 and T2 rotations in this study. Similarly, the relative abundance of *Actinobacteria* (an oligotrophic phylum capable of decomposing refractory organic matter under nutrient stress [62]), was higher in the rotation treatments (T1 and T2). The increased abundance of these phyla is consistent with findings that they positively correlate with crop health [63] and that *Actinobacteria* contributes to soil bacteriostatic effects [64]. These results align with other studies reporting that rotation increases the abundance of *Actinobacteria* and *Firmicutes* [32,65]. Finally, the inconsistent findings: contrary to reports that organic amendments increase the abundance of *Myxococcota* [66] and that rotation leads to higher *Myxococcota* abundance than continuous cropping [24], both the T1 and T2 rotations in this study showed a lower abundance of *Myxococcota*.

For the fungal community, the 10 most abundant fungal phyla across all samples were *Ascomycota*, *Mortierellomycota*, *Basidiomycota*, *unclassified_k__Fungi*, *Chytridiomycota*, *Zoopagomycota*, *Rozellomycota*, *Kickxellomycota*, *Olpidiomycota*, and *Blastocladiomycota*. This finding is consistent with the report by Shi et al. [31]. *Ascomycota*, *Basidiomycota* and *Chytridiomycota* predominant saprotrophic soil fungi capable of degrading aerobic cellulose, a process that improves soil fertility but can also increase disease incidence during the decomposition of organic matter [67]. The dominance of *Ascomycota* aligns with that of previous studies, identifying it as the predominant soil fungal phylum and a key driver of soil nutrient cycling [68]. In this study, the relative abundance of *Ascomycota* was significantly higher in both the T1 and T2 rotations compared to the CK treatment. In contrast, the relative abundances of *Basidiomycota* and *Mortierellomycota* were significantly lower in the T1 and T2 rotations than in CK. The relative abundance of *Chytridiomycota* exhibited a divergent response, decreasing significantly by 37.23% in T1 while increasing by 27.87% in T2 compared to CK.

At the fungi genus level, the ten most abundant fungi across all samples were *Fusarium*, *Gibellulopsis*, *Mortierella*, *Podospora*, *Pyrenochaetopsis*, *Cladosporium*, *Alternaria unidentified*, *Plectosphaerella*, and *Lectera*. This distribution is consistent with findings reported by Shi et al. [31]. *Fusarium* was the most abundant fungal genus in all samples, accounting for 12.57% to 18.32% of the relative abundance. Several of these dominant genera are known phytopathogens. Specifically, *Fusarium* can cause potato *Fusarium* wilt and dry rot, while *Lectera* is associated with legume stem diseases [69,70]. In this study, both the T1 and T2 rotation treatments significantly reduced the abundance of *Fusarium* and *Lectera*, compared to the CK (continuous cropping) treatment. The abundance of *Alternaria* (a genus responsible for potato brown spot, early blight, and soybean black spot [71]), was higher in T1 but lower in T2, relative to CK. These results align with previous findings that continuous potato cropping fosters higher abundances of pathogenic genera like *Fusarium*, *Lectera*, and *Alternaria* compared to rotation systems [33,72].

The genus *Mortierella*, which was also highly abundant, plays a complex role in soil ecosystems. While it possesses strong cellulolytic capacity that can enhance soil nutrient availability [73], some species are phytopathogenic and can cause seedling death [74]. In this study, the relative abundance of *Mortierella* was significantly lower in the T1 and T2 rotations than in CK.

In summary, the reduction in the abundance of key pathogenic genera like *Fusarium* indicates that the T1 and T2 rotation regimes are associated with a lower risk of soil-borne fungal diseases and represent healthier soil conditions compared to continuous potato monoculture. Specifically, T2 not only enriched *Firmicutes* and depleted *Fusarium*, but also increased the abundance of microbial taxa known to solubilize P and mineralize organic N. These microbial changes align closely with the 19% yield increase and with the enhanced soil fertility, suggesting that the agronomic benefits of T2 arise from a synergistic improvement in nutrient availability and pathogen suppression. The combined evidence from chemistry and microbiology therefore provides a coherent mechanistic account for why T2 outperformed the continuous-potato control.

### 3.4. Relationships Between Microbes and Soil Chemical Parameters

Soil microbial communities are strongly influenced by soil environmental factors, as soil physical and chemical properties directly affect microbial survival and reproduction. These microbial activities, in turn, drive soil nutrient cycling and material flow, ultimately enhancing soil quality. Redundancy analysis (RDA) showed that pH and TN were primary factors correlated with the soil bacterial community structure, followed by AP, AK, AN, and OM. This finding is consistent with that of Wang et al. [33], who identified pH and AN as the main drivers of bacterial community changes. For fungal community, our study found that soil fungal community structure was mainly affected by pH, TN, AK, and AP. Wang et al. and Zhao et al. indicated that fungal community was mainly regulated by soil pH [11,75].

At the phylum level, specific correlations with soil properties were observed. The relative abundances of *Methylomirabilota* and *Acidobacteriota* were positively correlated with AK. *Firmicutes* abundance was positively correlated with AN, whereas *Proteobacteria* abundance showed a significant negative correlation with TN, AN, and AK. *Myxococcota* was negatively correlated with AN and AK. These findings align with Zhao et al. [32], who reported significant correlations between bacterial community distribution and soil OM, AN, AP, and AK. Regarding fungal phyla, the relative abundance of *Zoopagomycota* was significantly negatively correlated with pH and positively correlated with AK. No other major microbial phyla showed significant correlations with the measured soil properties. The relationships between microbe and soil parameters may be complex and might contribute to plant–soil microbe interactions.

In summary, the data from this study present a cohesive narrative of how the T2 rotation system enhances potato productivity. The rotation drove a synergistic improvement in the soil environment, simultaneously enriching nutrient availability (Section 3.2) and fostering a microbial community shift toward beneficial, plant-growth-promoting taxa and away from key pathogens (Section 3.3). These below-ground changes collectively enabled the significant gains in tuber yield and quality observed in Section 3.1. This integrated view confirms that the success of T2 is not attributable to a single factor but is the emergent property of improved soil chemistry and a suppressive rhizosphere microbiome working in concert.

## 4. Limitations of the Study

This study’s field trial was designed to reflect regional potato cultivation practices within a double-cropping system. The results discussed are based on the initial two-year rotation cycle, representing the first cycle with summer maize and soybean. Consequently, the observed effects likely capture the initial impact of introducing crop rotation and altered management. However, maintaining the same specific rotation annually could eventually lead to challenges such as the buildup of soil-borne pathogens or a decline in yield. To address this, our future work will incorporate a wider range of local crops (e.g., sweet potato, summer peanut, mung bean, oilseed rape, winter wheat, and millet) and more diverse and extended rotation sequences. The objective of these long-term trials is to evaluate the effects of diversified crop rotations, potentially combined with other agronomic measures, on soil fertility and the microbial community. This will allow for the screening of optimal alternative rotation regimes. We anticipate that this work will provide new insights and strategies for maintaining soil health and promoting the sustainable production of potato.

## 5. Materials and Methods

### 5.1. Site Description

The field experiment was conducted during the period of 2023–2024 at the Agricultural Experimental station, Cotton Research Institute, Shanxi Agricultural University, located in Yanhu district, Yuncheng city, Shanxi province, China (N 35°09′, E 110°95′). The climate at the site is characterized as a temperate monsoon, with an average annual temperature of 13.6 °C, an average annual precipitation of 559.3 mm, an annual average sunshine duration of 2247.4 h, and a frost-free period of about 208 days a year. The soil type is cinnamon soil with a silty clay loam texture (18.0% clay, 27.0% sandy soil, and 55.0% silty sand). The initial basic topsoil layer (0–20 cm) properties was as fellows: pH 7.88, organic matter (OM) 31.3 g/kg, total nitrogen (TN) 0.93 g/kg, available nitrogen (AN) 20.5 mg/kg, available phosphorus (AP) 25.2 mg/kg, and available potassium (AK) 285 mg/kg.

### 5.2. Experimental Design

The field experiment used a completely randomized design with three replications. The size of each plot was 30 m^2^ (3 m × 10 m). The study included the following three treatments:

Continuous potato monoculture (CK): Potato was cultivated in a ridge-and-furrow system with plastic film mulching. A single row was planted on each ridge with a row spacing of 90 cm and a plant spacing of 21.5 cm, resulting in a planting density of approximately 51,750 plants per hectare. The early-maturing potato variety ‘Feiwuruita’ was planted from late February to early March and harvested in early June, after which the land was left fallow. Potato–summer maize rotation (T1): Potato was planted following the same pattern as the CK treatment. After the potato harvest, summer maize (variety ‘Wodan 77’) was planted on the ridges at a density of 67,500 plants per hectare. After the maize harvest, the straw was mechanically shredded and incorporated into the soil. Potato–summer soybean rotation (T2): Potato was planted following the same pattern as the CK treatment. After the potato harvest, summer soybean (variety ‘Yundou 101’) was planted on the ridges at a density of 180,000 plants per hectare. After the soybean harvest, the straw was mechanically shredded and incorporated into the soil.

Potato tuber pieces were planted into soil at a depth of 10–12 cm depth on the ridges (40 cm in width and 20 cm in height) on 2 March 2024. After planting, the tubers were mulched with polyethylene film immediately. Base fertilizers in the form of urea, calcium supper phosphate, and potassium sulfate (80 kg N/hectare and 102 kg P/hectare and 105 kg K/hectare) were applied before planting for each treatment and 45 kg N/hectare was applied as top dressing at the early flowering stage. The potatoes were irrigated three times, at the seedling stage, the early flowering stage, and the enlargement stage, at a rate of 2250 t/ha each time. The early-maturing potato variety was ‘Feiwuruita’ and the virus-free seed potato was provided by the Shanxi Province Potato Virus-Free Center. Soybean variety ‘Yundou 101’ and corn variety ‘Wodan 77’ were provided by Cotton Research Institute, Shanxi Agricultural University. Field management measures were performed manually during each treatment.

### 5.3. Potato Yield and Nutrition Measurements

After harvesting on 5 June 2024, the tuber yield and its structural composition were assessed. A 5 m section from the central row of each plot was harvested. The total tuber yield was determined by fresh tuber weight and the yield structure was analyzed based on the number of tubers per plant and the average weight of a single tuber.

To evaluate marketability, tubers from each plot were sorted into three size categories by weight: large (>150 g), medium (50–150 g), and small (<50 g). The number and weight of tubers in each category were recorded to calculate the commodity rate (proportion of marketable tubers) and the percentage distribution for each size class.

For quality analysis, tuber dry matter content was determined by drying fresh tuber samples at 70 °C for 48 h to a constant weight. For the resulting dry matter, the following chemical components were quantified: total nitrogen (TN, the Kjeldahl method), total phosphorus (TP, colorimetry), and total potassium (TK, flame photometry). The contents of starch, vitamin C, crude protein, reducing sugars, and the dry matter rate were determined with fresh potatoes. The starch content in tubers was determined by the anthrone colorimetric method. Vitamin C (Reduced Ascorbic Acid, AsA) was determined by UV spectrophotometry. Crude protein (total protein) was determined by the Kjeldahl method. Reducing sugars were determined by a 3,5-dinitrosalicylic acid colorimetric method.

### 5.4. Soil Chemical Analysis

Following the potato harvest, ten soil subsamples were collected from the 0–20 cm depth layer in each plot using an auger. These subsamples were thoroughly mixed to form a homogeneous composite sample per plot. Each composite sample was then divided into two parts. One part was immediately stored at −80 °C for subsequent DNA extraction and microbial community analysis. The other part was air-dried, ground, and passed through a 2 mm sieve for the analysis of the soil chemical properties.

Soil chemical parameters were determined by the methods of Lu [76]. Soil pH was measured potentiometrically using a FE20 pH meter (Shanghai Leici Scientific Instrument Co., Ltd., Shanghai, China) with a soil-to-deionized water ratio of 1:2.5 (*w*/*v*). Soil organic matter (OM) was determined by the potassium dichromate (K_2_Cr_2_O_7_) oxidation method followed by spectrophotometric measurement (Shanghai Metash Instruments Co., Ltd., Shanghai, China). Soil total nitrogen (TN) was measured using the Kjeldahl method. Soil available nitrogen (AN) was determined by the alkaline hydrolysis diffusion method. Available phosphorus (AP) was extracted with 0.5 mol/L sodium bicarbonate (NaHCO_3_) and quantified by molybdenum blue colorimetry; Available potassium (AK) was extracted with 1 M ammonium acetate (NH_4_OAc) and measured by flame photometry using an AP1302 flame spectrophotometer (Shanghai Instruments Group Co., Ltd., Shanghai, China).

### 5.5. DNA Extraction and Illumina MiSeq Sequencing

The extraction of soil DNA from a 0.5 g freeze-dried soil sample was performed using the E.Z.N.A.^®^ Soil DNA Kit (Omega Bio-tek, Norcross, GA, USA), following the manufacturer’s instructions. The concentration and quality of the extracted DNA were checked using a NanoDrop 2000 spectrophotometer (Thermo Scientifc, Wilmington, DE, USA).

The hypervariable V3-V4 region of the bacterial 16S rRNA gene was amplified with the primer pair 338F (5′-ACTCCTACGGGAGGCAGCA-3′) and 806R (5′-GGACTACHVGGGTWTCTAAT-3′). The fungal internal transcribed spacer (ITS) region was amplified with the primer pair ITS1F (5′-CTTGGTCATTTAGAGGAAGTAA-3′) and ITS2R (5′-GCTGCGTTCTTCATCGATGC-3′). Polymerase chain reaction (PCR) amplification, library construction, and Illumina MiSeq sequencing for both bacterial and fungal communities were conducted by Biomarker Technologies Co., Ltd. (Beijing, China).

The raw sequencing data were processed using the QIIME2 pipeline (version 2023.9). Quality-filtered sequences were clustered into operational taxonomic units (OTUs) at a 97% similarity threshold using USEARCH (version 10.0). Taxonomic classification of bacterial 16S rRNA gene sequences was performed against the Greengenes database (version 13.5), while fungal ITS sequences were classified using the UNITE database (Release 8.0).

### 5.6. Statistical Analysis

Alpha diversity indices, including the Chao1, Shannon, and ACE for bacterial and fungal communities, were calculated within the QIIME2 environment. Beta diversity was assessed using principal coordinates analysis (PCoA) based on Bray–Curtis distances to visualize differences in microbial community structure among treatments. The relationships between microbial community composition (bacteria and fungi) and soil properties were examined using redundancy analysis (RDA) with the ‘vegan’ package in R software (version 4.3.1). These bioinformatic analyses were performed on the free online platform of Beijing Biomaike Biotechnology Co., Ltd. (Beijing, China).

Prior to statistical analysis, the normality of the data distribution was confirmed with the Lilliefors test. One-way analysis of variance (ANOVA) with Duncan’s multiple range test (*p* < 0.05) was conducted using IBMSPSS Statistics (v.27.0) software (IBM Corp, Armonk, NY, USA) and Duncan’s multiple range test was performed using the least significant difference (LSD) to test for significant differences between treatments, with significance defined at *p* < 0.05. Pearson’s correlation analysis was performed to evaluate the relationships between microbial community parameters using Origin (v.2021, OriginLab Corp, Northampton, MA, USA). Figures were finalized using Adobe Illustrator (v.2021).

## 6. Conclusions

The potato–soybean rotation with soybean straw incorporation and potato–corn rotation with corn straw incorporation were effective strategies for enhancing potato productivity and soil health. These rotations significantly improved key soil chemical properties, including organic matter (OM), available phosphorus (AP), available potassium (AK), and total nitrogen (TN), while reducing soil pH. Consequently, tuber yield and quality were significantly increased.

*Proteobacteria*, *Actinobacteriota*, *Acidobacteriota*, *Chloroflex*, *Firmicutes*, and *Myxococcota* were predominant bacterial phyla and *Ascomycota*, *Mortierellomycota*, *Basidiomycota*, and *Chytridiomycota* were predominant fungal phyla. Soil bacterial community diversity was not affected, but potato–maize rotation significantly decreased the Chao1 index and the ACE index measures of soil fungal community diversity. These rotation regimes positively altered the soil microbial community structure. A notable beneficial effect was the suppression of pathogenic fungi, specifically reducing the abundance of *Fusarium*, *Alternaria*, and *Lectera* spp. The soil bacterial and fungal community structures were mainly affected by soil pH and total nitrogen. Overall, the findings demonstrate that integrating specific crop rotations with straw incorporation is a viable approach to maintain soil health and promote the sustainable production of potatoes.

## Figures and Tables

**Table 1:** The potato tuber yield and commodity rate in the different potato rotation treatments.

Treatments	Number of Tubers/Plant	Tuber Weight (kg/Plant)	Commodity Rate (%)	Large Tuber Rate (%)	Medium Potato Rate (%)	Small Potato Rate (%)	Tuber Yield (Tons/Hectare)
T2	10 ± 0.15 a	1.10 ± 0.03 a	82.00 ± 1.42 b	28.00 ± 0.75 a	54.00 ± 0.67 b	18.00 ± 1.42 b	53.52 ± 0.10 a
T1	9 ± 0.40 a	1.00 ± 0.08 a	88.89 ± 1.24 a	26.67 ± 0.56 b	62.22 ± 0.75 a	11.11 ± 1.24 c	52.50 ± 0.27 a
CK	7 ± 0.20 b	0.87 ± 0.02 b	74.28 ± 3.40 c	22.85 ± 2.10 c	51.43 ± 1.30 c	25.72 ± 3.40 a	44.35 ± 0.07 b

Note: The values indicate the mean ± the standard deviation. Different lowercase letters in a column indicate significant differences at *p* < 0.05. CK: Potato–potato rotation; T1: potato–summer maize rotation with maize straw incorporation; T2: potato–summer soybean rotation with soybean straw incorporation.

**Table 2:** Effects of potato rotations on potato quality indicators and dry matter.

Treatments	TN (g/kg)	TP (g/kg)	TK (g/kg)	Crude Protein (%)	Vitamin C (mg/g)	Starch (mg/g)	Reducing Sugar (mg/g)	Dry Matter (%)
T2	17.14 ± 0.57 a	1.47 ± 0.17 a	26.40 ± 1.45 a	10.71 ± 0.57 a	0.18 ± 0.01 a	150.1 ± 2.92 a	1.61 ± 0.01 b	18.84 ± 1.18 a
T1	14.07 ± 1.19 b	1.25 ± 0.12 ab	21.41 ± 2.32 b	8.79 ± 1.19 b	0.16 ± 0.01 ab	146.0 ± 6.65 a	1.64 ± 0.01 ab	17.45 ± 0.14 ab
CK	13.37 ± 1.01 b	1.09 ± 0.08 b	17.32 ± 0.63 c	8.36 ± 1.01 b	0.15 ± 0.01 b	113.3 ± 3.35 b	1.66 ± 0.02 a	16.69 ± 0.40 b

Note: The values indicate the mean ± the standard deviation. Different lowercase letters in a column indicate significant differences at *p* < 0.05. TN: Total nitrogen; TP: total phosphorus; TK: total potassium. CK: Potato–potato rotation; T1: potato–summer maize rotation with maize straw incorporation; T2: potato–summer soybean rotation with soybean straw incorporation.

**Table 3:** Soil parameters in different potato rotation treatments.

Treatments	pH	AN	AP	AK	TN	OM
		mg/kg	mg/kg	mg/kg	g/kg	g/kg
T2	7.74 ± 0.02 b	22.87 ± 0.73 a	28.41 ± 0.52 a	335.9 ± 5.53 a	1.08 ± 0.01 a	37.94 ± 0.26 a
T1	7.86 ± 0.01 a	21.12 ± 1.23 a	27.93 ± 1.54 ab	302.2 ± 2.52 b	0.97± 0.01 b	35.34 ± 0.38 a
CK	7.92 ± 0.02 a	20.50 ± 0.56 a	25.28 ± 1.16 b	285.5 ± 3.92 b	0.93± 0.01 b	31.85 ± 0.15 b

Note: The values indicate the mean ± the standard deviation. Different lowercase letters in a column indicate significant differences at *p* < 0.05. AN: Available nitrogen; AP: available phosphorus; AK: available potassium; TN: total nitrogen; OM: organic matter. CK: Potato–potato rotation; T1: potato–summer maize rotation with maize straw incorporation; T2: potato–summer soybean rotation with soybean straw incorporation.

**Table 4:** Alpha diversity indices of soil bacteria and fungi in different rotation treatments.

	Treatments	Chao1	ACE	Shannon
	T2	2874.22 ± 139.69 a	2880.81 ± 138.70 a	10.27 ± 0.04 a
Bacteria	T1	2706.17 ± 108.47 a	2712.43 ± 108.42 a	10.18 ± 0.04 a
	CK	2781.01 ± 140.72 a	2788.60 ± 140.26 a	10.22 ± 0.04 a
	T2	734.56 ± 46.61 a	741.80 ± 45.09 a	6.65 ± 0.06 a
Fungi	T1	665.59 ± 71.40 b	673.53 ± 70.41 b	6.19 ± 0.38 a
	CK	709.31 ± 21.86 a	715.47 ± 20.30 a	6.35 ± 0.20 a

Note: The values indicate the mean ± the standard deviation. Different lowercase letters in a column indicate significant differences at *p* < 0.05. CK: Potato–potato rotation; T1: potato–summer maize rotation with maize straw incorporation; T2: potato–summer soybean rotation with soybean straw incorporation.

## Data Availability

The data are contained within the article.
